# Reusing routine electronic health record data for nationwide COVID-19 surveillance in nursing homes: barriers, facilitators, and lessons learned

**DOI:** 10.1186/s12911-024-02818-3

**Published:** 2024-12-27

**Authors:** Y Wieland-Jorna, RA Verheij, AL Francke, R Coppen, SC de Greeff, A Elffers, MG Oosterveld-Vlug

**Affiliations:** 1 Nivel, Netherlands Institute for Health Services Research, Otterstraat 118, Utrecht, 3513 CR The Netherlands; 2 Tranzo, School of Social Sciences and Behavioural Research, Tilburg University, Tilburg, 5037 DB The Netherlands; 3 Amsterdam UMC, Vrije Universiteit Amsterdam, Department of Public and Occupational Health, Amsterdam Public Health Research Institute, Expertise Center for Palliative Care, Van Der Boechorststraat 7, Amsterdam, 1081 BT The Netherlands; 4 Department of Epidemiology and Surveillance, Centre for Infectious Diseases Control, National Institute for Public Health and the Environment, Postbus 1, Bilthoven, 3720 BA The Netherlands

**Keywords:** Electronic health records, Routinely recorded health data, Surveillance, Nursing homes, COVID-19

## Abstract

**Background:**

At the beginning of the COVID-19 pandemic in 2020, little was known about the spread of COVID-19 in Dutch nursing homes while older people were particularly at risk of severe symptoms. Therefore, attempts were made to develop a nationwide COVID-19 repository based on routinely recorded data in the electronic health records (EHRs) of nursing home residents. This study aims to describe the facilitators and barriers encountered during the development of the repository and the lessons learned regarding the reuse of EHR data for surveillance and research purposes.

**Methods:**

Using inductive content analysis, we reviewed 325 documents written and saved during the development of the COVID-19 repository. This included meeting minutes, e-mails, notes made after phone calls with stakeholders, and documents developed to inform stakeholders. We also assessed the fitness for purpose of the data by evaluating the completeness, plausibility, conformity, and timeliness of the data.

**Results:**

Key facilitators found in this study were: 1) inter-organizational collaboration to create support; 2) early and close involvement of EHR software vendors; and 3) coordination and communication between partners. Key barriers that hampered the fitness of EHR data for surveillance were: 1) changes over time in national SARS-CoV-2 testing policy; 2) differences between EHR systems; 3) increased workload in nursing homes and lack of perceived urgency; 4) uncertainty regarding the legal requirements for extracting EHR data; 5) the short notice at which complete and understandable information about the repository had to be developed; and 6) lack of clarity about the differences between various COVID-19 monitors.

**Conclusions:**

Despite the urgent need for information on the spread of SARS‐CoV‐2 among nursing home residents, setting up a repository based on EHR data proved challenging. The facilitators and barriers found in this study affected the extent to which the data could be used. We formulated nine lessons learned for developing future repositories based on EHR data for surveillance and research purposes. These lessons were in three main areas: legal framework, contextual circumstances, and quality of the data. Currently, these lessons are being applied in setting up a new registry in the nursing home sector.

## Introduction

The spread of the severe acute respiratory syndrome coronavirus 2 (SARS‐CoV‐2), causing the disease COVID-19, started in December 2019 and rapidly became a pandemic [1, 2]. The large number of COVID-19 patients confronted countries with challenges in healthcare provision [3]. There were numerous challenges in terms of information about the pandemic as well, for example regarding the number of citizens infected by the virus, the course of the disease, and the occupation of hospital beds. Such information is crucial for the adequate allocation of scarce resources on a regional, national, and global level. To acquire this information there was an acute and urgent need for datasets that are complete, accurate, and up to date [4, 5].

Older people in particular appeared to be at high risk of suffering from a severe form of COVID-19 [1, 6] and SARS‐CoV‐2 spread rapidly among residents in Dutch nursing homes [7, 8]. In the Netherlands, nursing home residents are mostly frail older people (Table 1). At the beginning of the pandemic in 2020, regional and national information on the spread of the virus in Dutch nursing homes was limited and had shortcomings. The Dutch Safety Board concluded in 2022 that this had resulted in an underestimation of the impact of the virus in nursing homes [9].

**Table 1:** Characteristics of the Dutch nursing home setting

In the Netherlands, older persons who need continuous high-level care or supervision can be admitted to a nursing home. Nursing homes provide care to older people with multiple and complex care needs that cannot be fulfilled by home care [10]. A specialized elderly care physician (in Dutch: specialist ouderengeneeskunde) is responsible for the medical care of nursing home residents. Elderly care physicians are certified after a three-year specialty training program in medicine for older adults, in addition to their basic university training as a medical doctor [11, 12]. In the year 2019, approximately 115,000 persons lived in a nursing home, almost three quarters of whom were female. The mean age of nursing home residents was 85 years in 2019 [13].	

In the absence of a national surveillance system in nursing homes, data routinely recorded by healthcare professionals in the electronic health records (EHRs) of nursing home residents could form the basis for a nursing home surveillance system without imposing an extensive additional administrative burden on healthcare providers. This is not simple to set up: several steps need to be taken to make routinely recorded health data fit for surveillance purposes.

Firstly, it is essential to gather information on the context in which the data is recorded. For example, in the case of COVID-19, it is crucial to know if and how susceptions of COVID-19 and a positive SARS-CoV-2 test result are recorded in the EHR. Secondly, data must be extracted from the EHR and transferred to the repository. In doing so, applicable legislation should be taken into account. Furthermore, as the EHR data was received from different systems and organizations, it is essential to assess its interoperability. Thirdly, the extracted data needs to be prepared into a dataset for researchers. Finally, the data file has to be shared with researchers to perform surveillance analyses Each step involves several choices and factors that can either facilitate or hinder the quality of the resulting data [14]. Therefore, setting up a repository requires careful consideration of these steps and factors to ensure the fitness of the data for surveillance and research purposes.

During and after the COVID-19 pandemic, several studies examined the quality and fitness of EHR records to identify COVID-19 patients [15–18] and the requirements for developing a COVID-19 registry [19–21]. However, none of these studies have specifically focused on the situation in Dutch nursing homes. This gap poses challenges when attempting to establish a repository and enhance pandemic preparedness in the nursing home sector. Therefore, this study describes the lessons learned regarding the attempt to develop a COVID-19 repository based on EHR data from Dutch nursing homes.

### COVID-19 repository for surveillance in Dutch nursing homes

To achieve a surveillance system for Dutch nursing homes, a data flow from EHRs into a COVID-19 repository needed to be developed in a short period. The development of the COVID-19 repository was initiated in March 2020 by the Dutch association for elderly care physicians (Verenso) and an umbrella organization for care providers for older adults and patients with a chronic disease (ActiZ), in collaboration with the Dutch Ministry of Health Welfare and Sport, EHR software vendors, legal experts, and researchers from the Netherlands Institute for Health Services Research (Nivel) and the Dutch National Institute for Public Health and the Environment (RIVM). EHR data were received between mid-May and the end of December 2020. The repository was set up within the program “Learning from Data” (2019–2024), carried out by a consortium consisting of UNO Amsterdam (University Network of Organizations for Care for Older Adults), the association of elderly care physicians Verenso, and the Nivel research institute. This endeavor was commissioned by RIVM. Nivel and RIVM were jointly responsible for determining and improving the quality of the data, the analyses, and publications based on the data. Data management was Nivel’s responsibility. The consortium aimed to provide national and regional insight into the course of the disease and the number of suspected and confirmed COVID-19 infections in nursing homes.

The EHR data collected by the COVID-19 repository were recorded in a standardized COVID-19 form in the nursing homes’ EHR systems. Some nursing home organizations used a different format. Table 2 describes the variables included in the form and the additional EHR data items that were collected.

**Table 2:** EHR data collected by the COVID-19 repository

• Verenso and one of the EHR software vendors developed a standardized COVID-19 form based on a template of openEHR [22]. • The form included patient information on: ◦ SARS‐CoV‐2infections (whether COVID-19 diagnostics have been started and whether COVID-19 is suspected, confirmed, or excluded); ◦ Follow-up information (whether a patient has recovered, shows clinical improvement or deterioration, has been admitted to a hospital, and has died in a nursing home or hospital). • The COVID-19 repository collected data recorded in the COVID-19 form and additional general background information on the nursing home resident (age and sex) and on the nursing home location (total number of male/female residents, type of nursing home care ward). • A pseudonymization tool developed by the trusted third party ZorgTTP [23] pseudonymized the data before the data were sent to the COVID-19 repository for storage.	

In December 2020, the consortium decided to discontinue the development of the repository and stop collecting data as of the end of that month. Several reasons contributed to this decision. An important reason was that, at that time, data from the SARS‐CoV‐2 tests administered by municipal health authorities were deemed more suitable to monitor the national spread of the virus as the testing policy applied to everyone suffering from COVID-19 symptoms [24]. Although the process of establishing the COVID-19 repository for nursing homes was discontinued, various lessons were learned that can inform the development of future repositories based on EHR data for surveillance and research purposes.

This article aims to describe the lessons learned regarding the development of the COVID-19 repository and the fitness of EHR data for the purpose of surveillance and scientific research. These lessons can be used for the development of new surveillance and research repositories based on EHR data and to improve pandemic preparedness in the future. The research questions addressed in this paper are:

- Which facilitators and barriers were encountered during the development of the COVID-19 repository for nursing homes with regard to: 1) legal requirements; 2) contextual circumstances (e.g. other repositories and surveillance initiatives, and recruitment aspects); and 3) data quality?
- What lessons can be learned for the development of future repositories based on EHR data for the purpose of surveillance and scientific research to ensure fitness for purpose?

## Method

### Study design

To answer the research questions, we performed an inductive content analysis [25] of documents that were written or receivd between March 2020 and February 2021 by the authors of this paper, all of whom were involved in the development of the COVID-19 repository. Furthermore, YWJ, MGOV, and AE evaluated the quality of the data that the repository received between May 2020 and December 2020. The results of the documentation and data quality analysis were sorted along the zones of the TRANSFoRm zone model.

### Analysis of documents

#### Eligibility criteria

The documents used in the analysis were selected based on the following inclusion criteria:

1. The document pertained to the COVID-19 repository.
2. All types of documents are eligible for inclusion. This included meeting minutes, emails, notes from phone calls with stakeholders (e.g. consortium members, contact persons of the seven largest EHR software vendors, boards of directors of nursing home organizations, elderly care physicians, and legal experts), and documents developed to inform stakeholders (e.g. website content, data extraction specifications, and legal information).
3. The document was written and saved between March 2020 and February 2021.

A document was excluded if it did not contain new information on the COVID-19 repository, such as an email expressing gratitude (e.g. “Thank you for the information”). Duplicates were also excluded.

#### Data analysis

Based on the eligibility criteria, 325 documents were selected. Inductive content analysis was conducted using MAXQDA 2022. Initially, two authors (YWJ and MGOV) read through the documents to get familiar with the content. Subsequently, codes were assigned to meaningful text units that addressed the research questions, focusing on factors facilitating or hindering the development of the repository. To ensure the reliability of the coding procedure, a considerable number of documents were coded independently by the first author (YWJ) and the last author (MGOV). Any disagreements were resolved by discussion. Finally, the two authors (YWJ, MGOV) collaborated to group the codes in a search for common themes.

#### Sorting themes along the TRANSFoRm zone model

**Fig. 1:**
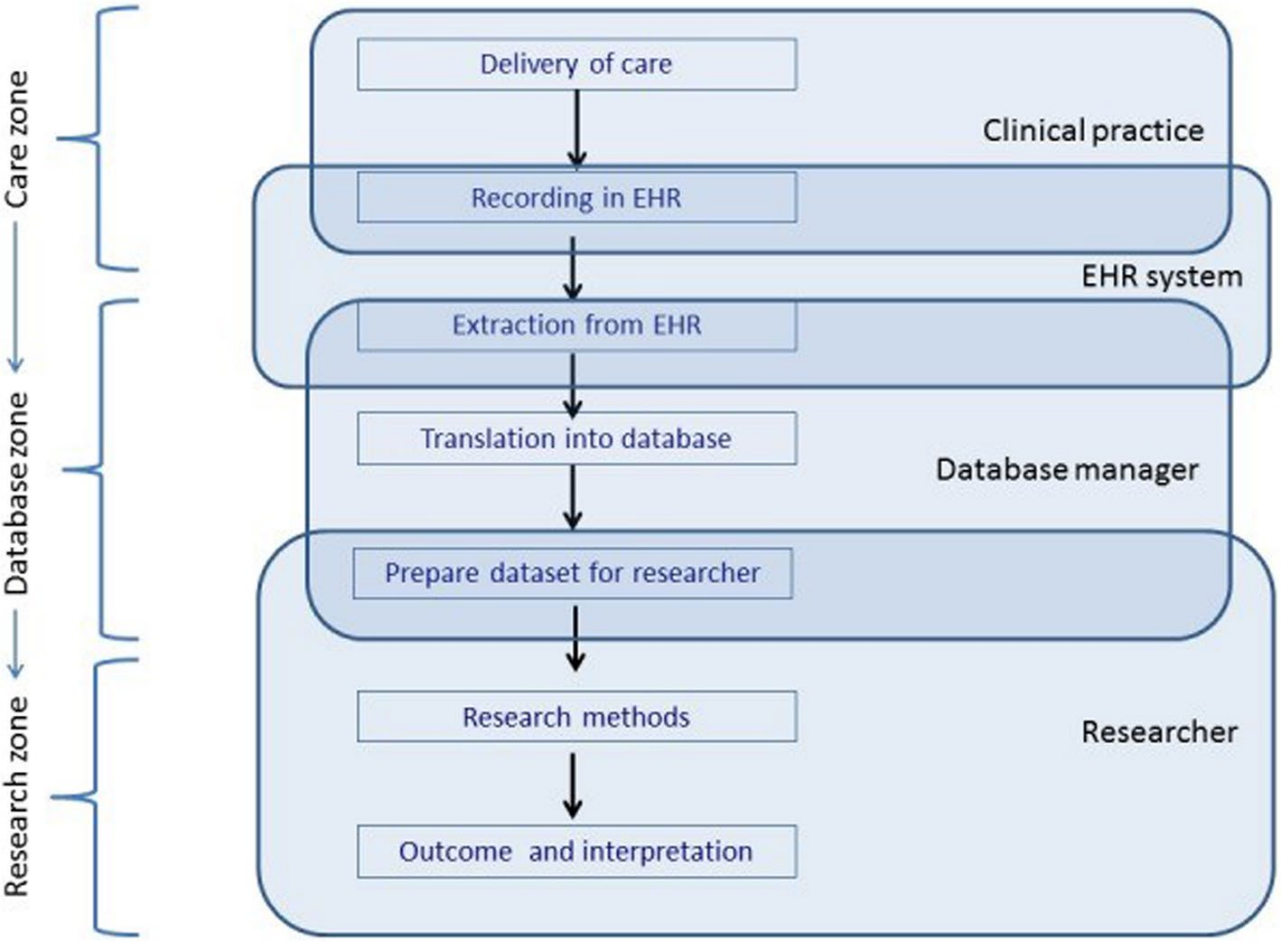
Steps and actors involved in the data flow from data recording in an electronic health record to the reuse of the data as described by Verheij et al. (2018) [14]

The results of the document analysis were sorted along the three zones of the TRANSFoRm zone model [22]. This model outlines the steps [14] involved in the reuse of routine EHR data for research which need to be taken to arrange the flow of EHR data from the initial recording of a clinically relevant event to applications processing the data and generating information (Fig. 1) [14]. Each step in this model involves several choices and factors that can ultimately affect the fitness of the resulting data for its intended use.

The first zone of the TRANSFoRm zone model (Fig. 1) is the “care zone” where delivery and recording of care take place with the primary aim of supporting healthcare provision and information exchange between healthcare professionals. Organizational aspects of the healthcare system, workload, healthcare policies, and guidelines have a big influence on what care is delivered and recorded. In the second zone, the “database zone,” data are extracted from an EHR, transferred to a database, and prepared into a dataset for researchers. The sharing and processing of health data must comply with the applicable legislation, posing potential challenges for the data flow. In the third zone, the “research zone,” researchers receive a research data file derived from the data in the database, which they can use to perform analyses [14]. The variety of challenges in each step may introduce bias and affect the extent to which data are fit for a certain purpose, such as for a surveillance system [26]. Therefore, conducting a data quality assessment is crucial to gaining insight into data-related issues [27].

For each theme identified in the inductive content analysis, the first author (YWJ) and the last author (MGOV) categorized it into one of the three zones of the TRANSFoRm zone model to understand its origin.

### Analysis regarding data quality

A data quality assessment provides insight into whether data are suited for a particular application (i.e. fitness for purpose) [26]. We used the commonly used framework developed by Kahn et al. (2016) [28] to explore the quality of the raw datasets as originally received from EHR software vendors or nursing homes. The framework of Kahn et al. (2016) covers three categories (conformance, completeness, and plausibility) [28], which are described below. In addition, we included ‘timeliness’ as a category in the data quality exploration. Based on the findings on the quality of the data, raw datasets were cleaned up (e.g. duplicates or non-plausible values were deleted) to give cleaned datasets.

#### Conformance

Conformance includes indicators for whether data values conform to requested constraints and specifications, enhancing interoperability and standardization among data suppliers [28]. We determined whether the data extractions were in line with a set of predefined specifications for the variables collected by the repository (Table 2). For example, whether a date variable had the requested format “dd-mm-yy”. This set of specifications was described in a document that was shared and discussed with the sending parties, which were in most cases the EHR software vendors. EHR software vendors used this set of specifications to extract data from the nursing homes’ EHR systems. If there were discrepancies between the specifications and the data we received, we asked the EHR software vendors for clarification. Where possible and if it was relevant for other EHR software vendors, we refined or clarified the set of specifications to increase the conformance of the data values. The revised document was shared directly with the EHR vendors and published online.

#### Completeness

Completeness refers to whether data values are present [28]. In the case of the COVID-19 repository, completeness of data is needed to provide a valid insight into regional and national trends. For each data file that the repository received, we explored completeness in two ways. First, on variable level. For example, whether required data variables, as described in the set of specifications, were included in the dataset and whether there were missing values for the required variables. Second, we monitored whether the data in the repository covered the various regions of the Netherlands.

#### Plausibility

A data value is plausible if it is likely to represent the real situation. Plausibility refers to the truthfulness of the value and therefore contributes to the accuracy of the data [28]. An example in the COVID-19 repository is that follow-up information should have a logical time sequence; e.g. when a nursing home resident has died, he or she cannot be “recovered” two weeks later.

#### Timeliness

As the repository aimed to provide real-time insight into the spread of SARS-CoV-2 among nursing home residents, we explored the timeliness of the data by determining the time between the initial point in the care zone where the data were generated, to the outcome and interpretation after the data had been analyzed by the researchers.

## Results

### Participants

**Fig. 2:**
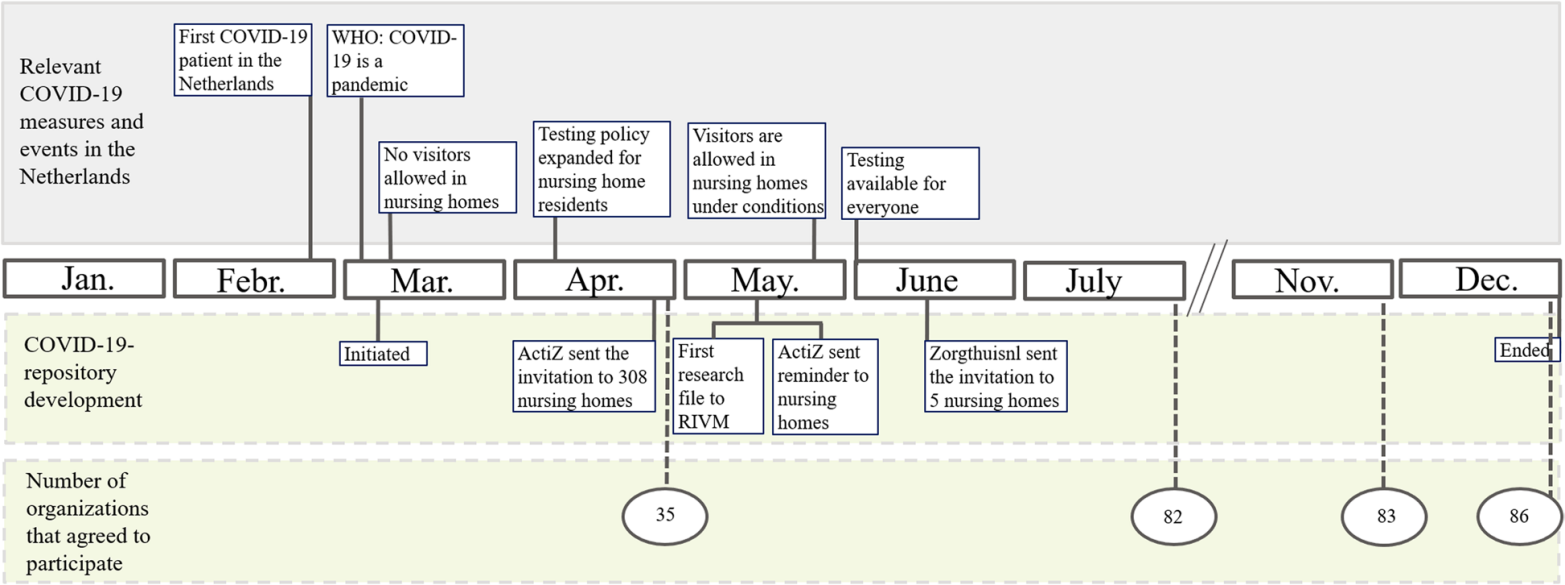
Timeline for 2020 showing COVID-19 measures in nursing homes and the development of the COVID-19 repository

An invitation to participate and provide data was sent to all 308 members (nursing home organizations’ boards of directors) of the umbrella organization ActiZ in April 2020. A reminder was sent to them in May 2020. A smaller umbrella organization (Zorgthuisnl) invited five additional nursing home organizations’ boards of directors in June 2020. Almost all nursing homes in the Netherlands are members of one of these two umbrella organizations. Figure 2 shows the timeline of the number of organizations that agreed to participate, the development of the COVID-19 repository, and the events and measures that were relevant for the COVID-19 repository development. At the end of April 2020 – a week after the first invitation was sent – 35 of 308 (11.36%) nursing home organizations expressed their intention to participate. In the months that followed, the percentage participating organizations increased to 26.19% (82 of 313 organizations), in July and to 27.47% (86 of 313 organizations) just before the COVID-19 repository was discontinued on 31 December 2020.

Table 3 shows the numbers of nursing home residents and nursing home organizations, and the number of different EHR systems from which we received data, included in the raw and cleaned datasets. The raw dataset included data values as originally received from the sender (i.e. EHR software vendor or nursing home organization), whilst the cleaned dataset is a refined version of the raw dataset. The raw dataset was cleaned up (e.g. duplicates or non-plausible values were deleted) based on the findings of the data quality evaluation. For example, the number of organizations included in both the raw and cleaned dataset increased from 15 organizations in May 2020 to 30 organizations in September 2020. In November and December, the raw dataset included 36 and 64 organizations, respectively. However, we had to clean the data resulting in 31 organizations in November and 35 in December due to implausible data values for the variable “nursing home organization”. This variable was filled with locations instead of organizations in the dataset received from one EHR software vendor. The EHR software vendor was informed about this finding and asked to revise their data extractions to comply with the predefined specifications. However, as it was not feasible for the EHR software vendor to revise the December dataset, all data from that vendor were removed from the cleaned dataset of December to avoid an overestimation of the number of organizations included.

**Table 3:** COVID-19 repository: number of nursing home organizations, residents and EHR systems included in the raw and cleaned datasets

	**Number of nursing home organizations included in:**		**Number of residents with a COVID-19 form included in:**		**Number of different EHR systems included in:**	
**Reference date on which the dataset was received**	Raw dataset^a^	Cleaned dataset^b^	Raw dataset^a^	Cleaned dataset^b^	Raw dataset^a^	Cleaned dataset^b^
05/15/2020	15	15	1343	1343	1	1
07/20/2020	27	27	3452	3452	1	1
09/14/2020	30	30	4418	4418	2	2
11/30/2020	36	31	7740	6998	4	2
12/28/2020	64	35	9318	8597	4	3

^a^Dataset as originally received from the source (i.e. EHR software vendor or nursing home)

^b^Modified dataset based on the findings of the data quality assessment

Furthermore, Table 3 shows that the number of nursing home residents for whom a COVID-19 form had been filled out increased over time. In the cleaned dataset, the number increased from 1343 residents in November to 8597 in December, as every week new cases were confirmed or suspected and the number of participating organizations increased. The number of EHR software vendors that were ready to extract and send data also increased during the repository’s existence. Until June, we received data files from only one EHR software vendor. After June, three more EHR software vendors were able to extract and send data files to the repository. In total, seven of the ten EHR systems operating in the nursing home sector, had integrated the COVID-19 form into the system and were willing to support the collection and sending of data to the COVID-19 repository. Although the exact percentage is unknown, these seven EHR software vendors provide an EHR system to the vast majority (estimated > 90%) of nursing home organizations in the Netherlands [29].

### Facilitators and barriers

The following sections describe the facilitators and barriers we encountered during the development of the COVID-19 repository, by using the framework of the TRANSFoRm zone model as described in the method [30].

### Care zone

#### Changes in national SARS‐CoV‐2 testing policy

The COVID-19 repository data collection took place during a period with important changes in the national SARS‐CoV‐2 testing policy. In the early months of the pandemic, testing capacity for individuals experiencing COVID-19 symptoms was not sufficient to test everyone. Later on, the Dutch government expanded the testing capacity (Fig. 2). Testing for nursing home staff became available on April 6, 2020, followed by easier access for nursing home residents from April 10, 2020 [31]. However, if COVID-19 was confirmed for two or more nursing home residents on a ward, all other residents with symptoms were recorded as “confirmed” without testing [32]. In June 2020, testing became available for everyone experiencing COVID-19 symptoms [33]. While increased testing was perceived as a facilitator for improving the quality of the EHR data on COVID-19, the dynamic changes in the national testing policy was perceived as a barrier. As the changes were reflected in the EHR data, the number of confirmed and suspected COVID-19 patients in nursing homes had to be interpreted within the context of the testing policy applicable at that particular moment. Therefore, it was a barrier for analyzing trends in the number of infected nursing home residents over time.

#### Use of several different electronic health record systems within the sector

Most nursing home organizations recorded COVID-19 information in their EHR system. To enhance standardization across EHRs, Verenso and one of the EHR software vendors developed a COVID-19 form (Table 2). In the Netherlands, nursing home organizations can choose between various EHR systems. The repository’s consortium reached out to vendors of the seven EHR systems with the largest market share in the nursing home sector, covering nearly all nursing home residents in the Netherlands. All seven EHR vendors made the COVID-19 form available in their system. However, variations in the design of the COVID-19 form implemented in the EHR systems may have influenced the completeness, conformity, and plausibility of the recorded data. For example, in one EHR system, a pop-up screen appeared to the physician every time the terms “COVID” or “Corona” were recorded in the EHR [32], whereas other systems did not have such a feature. This pop-up screen may have increased the number of filled-out COVID-19 forms compared to EHR systems without this feature. Hence, while a standardized form to record information and a pop-up screen in the EHR system are facilitators for collecting EHR data that are fit for the purpose of surveillance, differences in implementation between EHR systems could be regarded as a barrier as they may reduce the completeness and comparability of EHR data from different systems.

#### Perceived urgency of recording COVID-19 information in a standardized manner in the EHR system

To ensure complete insights, all elderly care physicians needed to record the COVID-19 information in the EHR. However, some nursing home organizations informed us that not all physicians in their organization filled out the COVID-19 form. One reason mentioned was that some physicians preferred to use another field in the EHR system because the COVID-19 form did not comply with their usual recording procedure. A large proportion of the nursing home staff experienced an increased workload due to increased care demands from infected residents and increased sick leave among the nursing home staff. Therefore, some physicians prioritized providing care over adapting their recording practices to comply with a standardized approach. The variation over time in the number of elderly care physicians using the COVID-19 form, both between and within organizations, posed a barrier to the fitness of the data for use in the COVID-19 repository.

### From care zone to database zone

In Europe, the collection of personal data is subject to the European General Data Protection Regulation (GDPR) and national legislation on this matter. Requesting explicit informed consent is often seen as the gold standard in the GDPR, but the GDPR allows for additional member-state legislation involving options other than explicit informed consent. The consequences of the GDPR and national legislation for the COVID-19 repository and surveillance were, however, not straightforward. Therefore, the first challenge in setting up the data flow from the care zone to the database zone was to determine the right legal basis for processing the EHR data and transferring the data to the database zone. A group of experts, including legal experts from the Dutch Ministry of Health Welfare and Sport, ActiZ, Verenso, Nivel, and an external law firm, were closely involved in exploring the possible legal requirements for the COVID-19 repository. In the following sections, we first describe the facilitators and barriers related to the legal requirements. Next, we describe other facilitators and barriers encountered during these steps.

#### Asking all nursing home residents for explicit consent cannot reasonably be expected in an urgent situation

In the case of the COVID-19 repository, the rapid spread of SARS‐CoV‐2 in nursing homes had placed an additional burden on the nursing homes’ staff, as a large number of residents were infected. Setting up an opt-in procedure that required asking all individual nursing home residents for explicit informed consent was expected to increase the burden on residents and staff even more. The consortium expected that nursing home organizations would refrain from participating in the COVID-19 repository if they had to ask all residents or their legal representatives for explicit consent. Another challenge was that the closure of nursing homes for visiting relatives made it difficult to get in touch with the legal representatives in cases where residents themselves were unable to provide explicit consent or incapable of doing so. Because of the urgent situation and its burden on healthcare professionals and patients, it was argued that asking all nursing home residents for explicit consent could not reasonably be expected from healthcare professionals. Deviation from the explicit informed consent default required further analysis to find an applicable legislative basis. Several scenarios were explored for the COVID-19 repository; they are described below.

#### Uncertainty regarding the legal requirements for extracting data from electronic health records

The most obvious scenario would have been to fall back on the exception rule of Article 458 of the Dutch Medical Treatment Contracts Act (WGBO) in which – under certain conditions – the reuse of EHR data without explicit consent is allowed for research purposes in the public interest. This scenario is allowed if: 1) asking for consent is not reasonably possible, for example, if this would be too high a burden for the patient; or [2] if healthcare providers or others cannot be reasonably expected to carry out a procedure to ask patients for explicit consent. Additional requirements include the requirement that the intended reuse is for statistics or scientific research in the field of public health that cannot be conducted without the data and that adequate measures (e.g. data pseudonymization) are taken to protect the patient’s privacy. An important safeguard in this scenario is that patients are given the opportunity to object against the reuse of the data pertaining to the individual. However, because of the different points of view of the legal experts and the different interests and purposes of the stakeholders, partners differed in how they interpreted the legislation. Therefore, an unambiguous substantiation to justify applying the exception rule of Article 458 did not seem feasible in the short term.

An alternative scenario was to aggregate the data to create an anonymous dataset before leaving the care zone. However, a disadvantage of this scenario is that individual patients cannot be followed over time. For the COVID-19 repository, monitoring the course of the disease at the patient level was deemed necessary to adequately track the spread and course of the virus among nursing home residents. In addition, as the number of infected residents in some nursing home locations might be low, aggregation would not always lead to anonymous datasets. Therefore, the aggregation of the data did not fit the purpose of the surveillance system.

As a third and final scenario, the legal basis was found in the legal obligation of RIVM to prevent and combat infectious diseases as described in the Dutch Public Health Act [34] and RIVM Act [35]. As the commissioning client of the COVID-19 repository, RIVM was allowed to acquire data from nursing homes based on its legal obligation. Nivel was given the task of extracting the data from the care zone and transferring the data to the database zone as a Processor (Art. 29 GDPR), because it had taken up a similar role in the “Learning from Data” program, and because of its experience in other projects. Therefore, Nivel had the knowledge and infrastructure needed for this task.

#### Inter-organizational collaboration to create a broad base of support in the nursing home sector

The development of the COVID-19 repository was an inter-organizational collaboration. In addition to the three organizations involved in the “Learning from Data” consortium, many other organizations were engaged in setting up the repository, for example, the Dutch National Institute for Public Health and the Environment (RIVM), EHR software vendors, and legal experts. In addition, the COVID-19 repository was supported by umbrella organizations for care providers of older adults and elderly care physicians. This helped to gain the trust of nursing home organizations in the appropriate and ethical reuse of the data and to stress the necessity of the surveillance and was therefore a facilitator. The consortium, umbrella organizations, and the medical profession carefully considered how they could show their involvement and support. For example, it was decided to let umbrella organizations for older adult care providers inform nursing homes’ boards of directors about the COVID-19 repository and to stress that the COVID-19 repository valued the interests of nursing home organizations and their residents.

#### Early and close involvement of vendors of electronic health record systems

Because of the urgency of the pandemic, EHR software vendors were willing to speed up the developmental process. In addition, involving EHR software vendors in the early development of the data infrastructure, holding frequent meetings, and the accessibility of the vendors’ contact persons were facilitators for setting up the repository. However, there were also some barriers. First, EHR software vendors needed time to explore the technical and legal possibilities. Not all contracts between a nursing home organization and its EHR software vendor allowed data collection and sharing by the EHR software vendor, or the EHR software vendor did not want to take this responsibility. Additionally, in cases where the EHR database was managed by the nursing home organization (i.e. it was stand-alone), EHR software vendors faced challenges in collecting and sending data on behalf of the nursing home. Second, EHR software vendors questioned the pseudonymized method of the trusted third party to transform the unique identifier (i.e. the social security number) into a unique but irreversible and non-traceable identifier. They needed time to understand the pseudonymization method before they were convinced that the trusted third party could guarantee the privacy of the nursing home organizations and residents. Third, one EHR software vendor decided to build a tool that nursing home organizations could use to extract and send the data and faced unexpected delays in its development. Hence, the high dependency on the cooperation of EHR software vendors was a barrier in setting up the repository.

#### Coordination and communication between the consortium partners

The COVID-19 repository was developed within the context and funding of a broader infrastructural program that already existed before the COVID-19 pandemic occurred, namely the program “Learning from Data,” as the repository fitted with the program’s aims. The repository could thus benefit from the relationships between the parties involved in this program. However, due to the urgency of the pandemic, it was a challenge to prepare and make project-specific agreements between the consortium partners before they started developing the repository. These agreements included task allocation between Nivel and RIVM for receiving, sending, and analyzing the data, as well as agreements between organizations involved in informing and recruiting the nursing home organizations. Therefore, during the whole process in which the COVID-19 repository was set up, the consortium partners consulted each other frequently in digital meetings and by e-mail to make these agreements. Coordination and clear communication between consortium partners helped to work efficiently by making and refining project-specific agreements during the developmental stage.

#### Providing complete and understandable information to nursing homes’ boards of directors

All invitations sent to nursing homes included information on the legal framework and which health data would be reused and for what purpose, a description of the infrastructure including data pseudonymization, a form for instructing the EHR software vendor to send data to Nivel, and information that could be placed on the nursing home’s website to inform residents and their representatives. A challenge faced by the COVID-19 repository was the need to provide complete and easily understandable information at short notice. Furthermore, not all invitations were taken into consideration by the boards of directors, for example, because they simply did not read the e-mail. Therefore, the COVID-19 repository provided information through different channels, whereby the information could be adapted based on questions frequently asked by the nursing home staff and their boards of directors. This included articles in newsletters for elderly care physicians, social media messages focusing on a broad target group, and a website set up to provide information on the purpose of the data collection and reuse, variables included in the dataset, infrastructure, and frequently asked questions. Furthermore, nursing home staff could ask questions via e-mail or by telephone.

Once the invitation was sent out, a barrier for some organizations was that the agreement between the nursing home organization and the research institute Nivel, stating the specific rights and obligations of both organizations, was not included in the initial invitation. Instead, the nursing homes’ boards of directors were informed of the conditions that applied to the processing of data by Nivel. To speed up the process, the collection of data of nursing home organizations that had agreed to participate started before the agreement was signed. However, a few organizations decided to postpone sharing data until they had received and signed the contract.

#### Prioritizing care provision over participating in a surveillance

Not all nursing home organizations were willing or able to prioritize their contribution to the COVID-19 repository. Some of the organizations that declined participation told us that they prioritized healthcare provision over administrative tasks because of the increased workload resulting from infected residents in their organization and the increased absence of sick employees. They expected that participation in the COVID-19 repository would further burden the healthcare professionals. In addition, the Dutch government decided to ease COVID-19 measures between June and August 2020, which reduced the incentive for nursing homes and the perceived urgency to participate.

#### The existence of multiple COVID-19 monitors raised questions

Due to the absence of a national surveillance system for nursing homes at the beginning of the pandemic, the COVID-19 repository was developed. However, as the pandemic evolved, other COVID-19 monitors were developed as well. For example, multiple regional collaborations monitored the spread, and the SARS-CoV-2 testing data from the municipal health services became more and more reliable as the national testing capacity was scaled up. This raised questions within the nursing home sector about how these monitors differed from each other, whether all of them were needed, and which monitor a nursing home should share its COVID-19 data with. Although the differences with other monitors were explained in the information provided to nursing homes, a significant barrier was that a considerable number of nursing home organizations had difficulties distinguishing the different initiatives. Some of these organizations thought that they participated in the COVID-19 repository, while in fact they provided data to another initiative. The Dutch Ministry of Health, Welfare and Sport supported the COVID-19 repository and wanted to incorporate the surveillance information into a national dashboard. However, nursing home organizations could not be forced to participate.

### From database to research zone

#### No up-to-date list of all nursing home organizations in the Netherlands

To monitor whether the repository contained data from a representative set of nursing homes, we needed an up-to-date and complete overview of all nursing home organizations in the Netherlands, including their different locations and numbers of beds. However, a barrier was that such an overview was lacking. Therefore, we had to combine different sources and search nursing home organizations’ websites to verify whether our information was correct.

#### Data quality requires improvement

As described in the Method section, we evaluated the quality of the EHR data for four categories: conformance, completeness, plausibility, and timeliness. During the period the repository was operable, much attention was paid to address data quality issues that arose during the flow of data from the care zone to the database zone, and finally to the research zone. Below, we describe for each category the main barriers and facilitators found for the COVID-19 repository.

First, we explored the conformance of all datasets with predefined specifications for the data values. The challenge here was to provide unambiguous specifications. Although we went through the specifications with the EHR software vendors, each EHR software vendor, database architect, or researcher interpreted the specifications based on their knowledge and experience. Especially in the beginning, we saw discrepancies between the data we received and the specifications we intended. Therefore, if discrepancies occurred, we asked EHR software vendors for clarification. We refined, where possible, the specifications or requested EHR vendors to comply with existing specifications. This close collaboration with the EHR software vendors was a facilitator to improve the conformance of the data values.

Second, we evaluated the completeness of the datasets for three levels: EHR system, regional, and national. At the EHR system level, a barrier to data completeness was that not all EHR systems could extract the requested set of data, either because there was no entry for the variable in the system or because the complex database structure hampered the extraction of the data values. Addressing this, we allowed to leave data values empty if the information could not be extracted. For example, this was the case for the type of nursing home care (e.g. somatic or psycho-geriatric). Another barrier was determining whether the total number of filled-out forms was complete, as it was unknown how consistently elderly care physicians used the COVID-19 form. Lastly, the completeness of the data at the regional and national levels was affected by the number of participating nursing home organizations. While in December 2020, 86 of the invited organizations were willing to share COVID-19 data, only 35 organizations were ready to share data with the COVID-19 repository. Unfortunately, the received data were not sufficiently complete to provide insight into the spread of COVID-19 in all regions in the Netherlands.

Third, we explored the plausibility of the data considering whether the data values were believable or truthful within the context of pre-existing knowledge. Implausible information in the data extractions was mainly caused by recording errors or settings of an EHR system. We were not able to fully overcome these barriers due to the dependency on EHR software vendors and the physicians recording the data. Recording errors were, for example, reflected by data values that did not change over time as expected. Another example of implausible data in the COVID-19 repository was a nursing home organization with more than 100 different locations in the dataset, while based on the nursing home’s website we knew that this was implausible. An additional barrier was the aforementioned lack of a comprehensive national overview of the number of different nursing home locations. Therefore, we had to search for information on nursing homes’ websites, which was time-consuming. Due to differences between EHR systems and different internal procedures in nursing homes, determining the plausibility of a value was a necessity for providing insight into the fitness of the data for surveillance purposes, and required information on the context in which the data were recorded and extracted.

Fourth, the timeliness of the data was determined from the initial point in the care zone, where the data were generated, to the outcome and interpretation after the data had been analyzed by the researchers. Between April and July 2020, the EHR software vendors and nursing home organizations sent data to Nivel three times a week – on Mondays, Wednesdays, and Fridays before 10 a.m. By using the extraction software developed by the EHR software vendor and the pseudonymization tool of the trusted third party, extracting and sending a dataset to Nivel required limited effort on the part of the sending party (i.e. nursing home organization or EHR software vendor). The frequency of three times a week was experienced as a barrier for delivering data to the repository for a significant number of nursing homes and some of the EHR software vendors, the frequency was reduced to once a week. After receiving the raw data sets, exploring the data quality, and deriving a researcher data set, Nivel sent it to RIVM the next day at the latest. Subsequently, researchers from RIVM explored the quality of the data and discussed the findings with Nivel researchers. RIVM, Nivel, EHR software vendors, and participating nursing home organizations agreed that the frequency could be increased if the number of COVID-19 patients in nursing homes rapidly increased again.

## Discussion

Based on an inductive content analysis, this study describes the facilitators and barriers encountered during the development of a repository for the purpose of COVID-19 surveillance in Dutch nursing homes. We used the TRANSFoRm Zone model [14, 36] to describe the steps taken before routinely recorded health data on nursing home residents could flow into the COVID-19 repository and identified facilitators and barriers in each of the three zones described in the model (care, database, and research zone). The results of this study show that the development of the COVID-19 repository was a complex process in which many barriers and facilitators are interrelated. For each facilitator or barrier we found, we determined if it was related to legal requirements, contextual circumstances, or to the fitness of the data for surveillance and research (Table 4). Based on our findings, we formulated nine lessons learned for setting up a repository using electronic health records for surveillance and research (Tables 4 and 5). The lessons learned include calls for action on the macro- (e.g. government and policymakers), meso- (e.g. EHR vendor, umbrella organizations), and micro level (e.g. project team of the surveillance system and medical professionals), and demand collaboration among a broad range of stakeholders (Table 5).

**Table 4:** Facilitators and barriers encountered during the development of a repository for pandemic surveillance and research based on electronic health records

**Facilitators and barriers found in this study**	**Finding relates to**			**Leads to lesson**
	Legal requirement	Contextual circumstance	Fitness for purpose	
**Care zone**				
Changes in national SARS‐CoV‐2 testing policy		x	x	2, 9
Use of several different electronic health record systems within the sector		x	x	2, 3, 5, 6, 9
Perceived urgency of recording COVID-19 information in a standardized manner in the EHR system		x	x	4, 7, 9
**From care zone to database zone**				
Asking all nursing home residents for explicit consent cannot reasonably be expected in an urgent situation	x	x	x	1, 4, 9
Uncertainty regarding the legal requirements for extracting data from electronic health records	x		x	1, 4, 9
Inter-organizational collaboration to create a broad base of support in the nursing home sector		x	x	4, 5, 9
Early and close involvement of vendors of electronic health record systems		x	x	2, 3, 5, 9
Coordination and communication between the consortium partners		x	x	5, 9
Providing complete and understandable information to nursing homes’ boards of directors	x	x	x	1, 2, 4, 6, 7, 8, 9
Prioritizing care provision over participating in a surveillance		x	x	2, 3, 4, 7, 9
The existence of multiple COVID-19 monitors raised questions		x	x	2, 4, 6, 9
**From database to research zone**				
No up-to-date list of all nursing home organizations in the Netherlands		x	x	8, 9
Data quality requires improvement		x	x	9

**Table 5:** Lesson learned for setting up a repository for pandemic surveillance and research based on electronic health records

**Lesson**	**Description**	**Level at which action should be initiated**	**Main stakeholder groups involved**
**Legal requirements**			
Lesson 1: Prepare and implement a legal framework for pandemic surveillance and research	Currently, national legislation concerning the collection of EHR data is unclear, which may limit the use of EHR data and require additional data protection measures. There are different rules and regulations, the applicability of which is under constant debate. Therefore, preparing for different scenarios, and if possible implementing specific legal requirements and adequate data protection measures, will speed up the time needed to make EHR data available for surveillance and research when the data are needed	Macro	• Government/ policymakers • Legal experts • EHR software vendors
**Contextual circumstances**			
Lesson 2: Ensure national coordination for reusing health data for the purpose of surveillance	Instead of the uncontrolled growth of many repositories, a national coordination body should ensure an efficient and effective flow of data from EHR systems to researchers analyzing the data. Before a new pandemic occurs, explore and describe how the coordination and the data flow could be organized in different situations	Macro	• Government or other national coordination body, and policymakers • Patients and medical professionals and organizations (e.g. represented by an umbrella organization) • Researchers and other data users • EHR software vendors
Lesson 3: Ensure national coordination for data standardization	National coordination and the involvement of relevant stakeholders are needed to set definitions about recording data on a (new) infectious disease and other routinely recorded EHR data to make the data interoperable. Implement these definitions in a standardized manner in EHR systems. Start with the standardization of basic information in nursing homes’ EHR systems	Macro	• Government or other national coordination body, and policymakers • Medical professionals and organizations (e.g. represented by an umbrella organization) • Researchers and other data users • EHR software vendors
Lesson 4: Work together with the medical profession, patients, relevant umbrella organizations, and other relevant stakeholders	Support from the medical profession, patients, relevant umbrella organizations, and other stakeholders is crucial to creating trust and awareness of the necessity of a surveillance system. Carefully consider and select the relevant organizations, and determine their roles and responsibilities in the surveillance	Meso	• Medical profession • Patients • Relevant umbrella organizations
Lesson 5: Ensure coordination and clear communication between the collaboration partners	The time to prepare and discuss workflows and procedures is limited when there is an urgent and acute need for complete, accurate, and up-to-date datasets. Coordination and clear communication between collaboration partners help to ensure an efficient workflow to reach goals jointly. In addition, close collaboration with EHR software vendors operating in the nursing home sector is essential, as the EHR is the initial source of data. Early involvement of EHR software vendors enables them to provide input for developing a user-friendly feature to record and extract data, and scheduling the development and implementation	Micro	• Project coordinator • Collaboration partners • EHR software vendors
Lesson 6: If possible, make use of an existing data infrastructure	As allocating expertise and resources (e.g. financing and technical expertise) to set up a new repository is difficult at short notice, connecting to a repository or data infrastructure that has already been developed may speed up the developmental process. However, such a repository should be sufficiently flexible to allow adjustment for the new form of data collection	Meso	• Government or other national coordination body, and policymakers • Repository or data infrastructure that already has been developed
Lesson 7: Prepare a recruitment strategy with sufficient time and resources	The surveillance system needs a recruitment strategy to recruit enough healthcare organizations to share data. First, adequate and understandable information on aspects including the aim, necessity, method of data processing, how to inform patients, and privacy safeguards needs to be developed and sent to healthcare organizations. Second, time and resources are needed to follow up on the healthcare providers that have been informed, for example, to check whether they have read and understood the information and whether the organization wants to participate	Micro	• Recruitment team • Patients and medical professionals and organizations (e.g. represented by an umbrella organization) • EHR software vendors
Lesson 8: Prepare a national, up-to-date overview of all nursing home organizations within a country	Develop a national overview with basic background information, such as the number of organizations and locations. Such an overview can already be developed and should be updated regularly regardless of whether there is a pandemic	Macro	• Government or other national coordination body, and policymakers • Medical professionals and organizations (e.g. represented by an umbrella organizations)
**Fitness for purpose**			
Lesson 9: Continuously assess and improve the quality of EHR data	Several steps need to be taken to make routinely recorded healthcare data fit for surveillance and wider research purposes. Facilitators and barriers, and choices made in each of these steps may affect the fitness of the data for surveillance purposes. Surveillance systems require data that are up to date and reliable. A data quality assessment helps to gain insight into the quality of the data. The data quality assessment is a continuous process that includes discussing the results with the source of the recorded data (e.g. medical professionals and EHR software vendors) to interpret the findings and improve the quality of the data	Micro	• Projectteam of the surveillance system • Medical professionals and organizations (e.g. represented by an umbrella organizations) • EHR software vendors

Below, we summarize the main lessons learned with regard to legal requirements, contextual circumstances, and data quality. The results of this study could help to improve pandemic preparedness in the future.

### Clarification of the legal requirements

A major challenge for setting up the COVID-19 repository for nursing homes was the search for legal requirements applicable to the extraction and reuse of health data from EHRs for the purpose of surveillance. Although international and national legislation allow the reuse of health data under certain conditions, determining the consequences for the COVID-19 repository was not straightforward. Exploring and describing the legal requirements for the repository, and discussing their relevance with stakeholders, was a time-consuming process and required the involvement of experts from different fields. An adequate basis to collect and reuse health data is needed not only to comply with the legislation, but also to gain trust in the appropriate reuse of the data. This trust is an important element for patients and organizations deciding to share health data for purposes beyond the use of health data for healthcare provision, such as research or surveillance purposes [37, 38]. In addition, regarding the lack of clarity of the legal requirements, the requirements and their consequences were weighed up against the interests of each of the consortium partners. This led in some cases to a different interpretation of the GDPR by some partners, which complicated the search for the legal requirements. For these reasons, our lesson learned is to provide clarity for different scenarios regarding the legal basis on which health data may be collected and which data protection measures are required (e.g. a consent procedure and data pseudonymization). This will speed up the time needed to make EHR data available for surveillance and research if necessary. Although we formulated only one lesson (Lesson 1 in Table 5) for implementing the legal requirements, this lesson is crucial to being able to reuse EHR health data for surveillance and research in an urgent situation. The legal requirements provide the foundation for the flow of data from the care zone to the database zone. This lesson is in line with other literature in which the lack of clarity concerning the legal requirements for the reuse of health data for research is mentioned as a barrier [39, 40]*.* At the macro level, it is strongly recommended to prioritize actions to remove this barrier.

### Take contextual circumstances into account

Most barriers and facilitators for the COVID-19 repository were found for contextual circumstances and occurred in all three zones of the TRANSFoRm Zone model. Examples are the changing national testing policy, confusion about the differences between various COVID-19 surveillance initiatives, and considerations related to the standardization of EHR records.

A key facilitator in stressing the necessity of surveillance, raising awareness, and to timely developing and implementing a data infrastructure to collect the data was the early engagement and support of relevant stakeholders on micro-, meso-, and macro level. These stakeholders include the medical profession, patients, umbrella organizations, and EHR software vendors (Lesson 4). In addition, this collaboration should be coordinated at the national (macro-) level (Lesson 2) and between the collaboration partners (micro level) (Lesson 5). For example, the government could take the lead in developing a national repository or delegate this responsibility to a specific body. Coordination might include only using this designated repository by parties monitoring the spread of a virus. In Europe, coordination is also influenced by the European Health Data Space (EHDS) which may be a step forward in preparing an infrastructure for future pandemics [41].

The developmental process could be shortened by connecting the new repository to an existing repository that is sufficiently flexible to allow adjustment to a new kind of data collection, such as a sentinel surveillance network (Lesson 6). During a pandemic, the increased workload on healthcare professionals may lead healthcare providers to prioritize care provision over getting informed over and involved in data-sharing initiatives for surveillance and research purposes. Therefore, one lesson learned on the level of the surveillance system itself (micro level) is to prepare and implement a recruitment strategy with ample time and resources to provide adequate and understandable information to all stakeholders and to follow up on a healthcare provider’s and patient’s willingness to participate and share data recorded in EHRs (Lesson 7). Previous studies showed that transparent, adequate, and understandable information increases patients’ willingness to share EHR data for research [42, 43] In addition, the information provided to the boards of directors and individual medical professionals who should record the information in the EHR is important. Literature on healthcare provider recruitment for research shows that interest in the aims of the research is important for organizations deciding to participate. Clear communication about the aim and necessity of the research and effort required by the healthcare provider is identified as a facilitator to persuade them to participate [44, 45]. Even though these studies did not focus on sharing EHR data for surveillance, we have noticed that these aspects hold true for the recruitment of healthcare organizations for the COVID-19 repository as well. Moreover, due to the urgent character of a pandemic and potentially increased workload, it may be even more important to set up an efficient recruitment strategy that clarifies the necessity and makes it easy to participate.

Another lesson learned with regard to contextual aspects is the importance of standardized definitions (initiated at the macro level) (Lesson 3). Here as well, the involvement of stakeholders on micro-, meso-, and macro level, including the medical profession, umbrella organizations, and EHR software vendors, and national coordination are very important. Standardized definitions could be defined and implemented as soon as possible to increase interoperability. Current initiatives, for example, the Dutch KIK-V program [46], are already working on this.

The influence of the contextual circumstances on the fitness of COVID-19 EHR data for the purpose of surveillance and research as found in this study, were recognized in other literature as well. For example, Stracci et al. (2023) describe, based on published studies, challenges in correctly interpreting and comparing datasets from hospitals. The challenges, such as changes in bed availability, admission policies, and level of population immunity introduced bias and limited the comparability within and between datasets [47]. Another example is the paper of Madhavan et al. (2020) in which the authors analyzed the experiences of 15 academic medical centers in the US and found barriers with regard to uncontrolled growth of data-sharing initiatives and lack of data standardization. Madhavan et al. (2020) conclude that a successful data-sharing infrastructure for COVID-19 and other diseases requires national coordination and collaboration between many stakeholders [5]. The importance of national coordination and working together with key stakeholders for the reuse of electronic health data before and during a pandemic is recognized in other studies as well [48–53]. Despite this recognition based on a long-run global history of pandemics (e.g. influenza and Ebola), the COVID-19 pandemic in the Netherlands showed that the nursing home sector was not in a position to rapidly share data on nursing home residents.

### Importance of a data quality assessment

Lastly, we formulated a lesson learned from the data quality assessment. Facilitators and barriers encountered during the flow of EHR data from the care zone via the database zone to the research zone are expected to be reflected in the quality of the EHR data. It is, therefore, important that the project team of the surveillance system (micro level) determines the quality of the EHR data from an early stage and continuously discusses the data quality with stakeholders, such as medical professionals and EHR software vendors. This helps to interpret the findings, to improve the quality of the data, and to create datasets based on electronic health records that are complete, accurate, and up to date in such a way that they are fit to be used for the purpose of surveillance during a pandemic (Lesson 9).

### Strengths and limitations

To our knowledge, this is one of the first studies describing the facilitators and barriers encountered during the development of a repository based on EHR data for surveillance purpose in the nursing home sector. A strength of our methodology is that we have analyzed documents that were written, saved, and used during the development of the COVID-19 repository, limiting the risk of recall bias. Through inductive content analysis, we systematically analyzed the documents, with the TRANSFoRm Zone model serving as a valuable tool for understanding the origins of the facilitating or hampering factors. This insight can inform strategies for improvement. The lessons learned in the Dutch nursing home sector can be used for setting up surveillance systems in other healthcare sectors and countries. Also, they align with other studies describing challenges in using EHR data on COVID-19 for surveillance and research purposes. In addition, these lessons might not only be relevant for repositories collecting EHR data for surveillance purposes but also for other types of research. For example, the lessons are currently being used to develop the Registry Learning from Data in Nursing Homes – a Dutch national registry of data from EHRs on care delivered by elderly care physicians in nursing homes to allow EHR data reuse for research and the improvement of the quality of care [54].

There are some limitations to consider. Firstly, although extensive information was documented during the repository’s development, some information might be missing. However, since researchers involved in the repository’s development were also engaged in this study, findings could be interpreted in the context of that time. Secondly, some of the authors of the documents reviewed in this study are also authors of this manuscript, which might have influenced the outcomes. However, the authors’ knowledge of the context of the development of the repository is helpful in interpreting the findings of this study. Furthermore, a substantial part of the documents comes from various other stakeholders including consortium members, contact persons of the seven largest EHR software vendors, boards of directors of nursing home organizations, elderly care physicians, and legal experts. Thirdly, future research on facilitators and barriers to reusing EHR data for a surveillance system in an international context is recommended. As countries may differ on how their public health systems and surveillance systems are structured, certain lessons may be more relevant in some contexts than others. Collaboration and data exchange between countries is also important for monitoring the spread of an infectious disease. Lastly, although the data quality framework provided valuable guidance, we did not measure data quality. As a repository is more evolved than the COVID-19 repository was, measuring data quality becomes more important, serving as a useful tool to monitor and detect unexpected changes in data quality.

## Conclusions

Acquiring complete, accurate, and up-to-date datasets is crucial in monitoring the spread of infectious diseases to detect trends and outbreaks. Health data from electronic health records can provide this information. However, several challenges may limit the fitness of EHR data for the purpose of surveillance and research. Based on facilitators and barriers encountered during the development of a COVID-19 repository for the purpose of surveillance in Dutch nursing homes, we have formulated nine lessons learned with regard to three themes: 1) legal requirements; 2) contextual circumstances; and 3) data quality. These lessons can help researchers, policymakers, medical professionals, and other stakeholders to be better prepared for setting up a repository to collect EHR data for the purpose of surveillance and research. Currently, these lessons learned are being applied in setting up a new registry in the nursing home sector.

## Data Availability

Data analyzed during the current study are not publicly available due to reasons of confidentiality. However, codes and themes are available from the corresponding author upon reasonable request.

## Abbreviations

EHR: Electronic health record
GDPR: General Data Protection Regulation
WGBO: Dutch Medical Treatment Contracts Act
